# Treatment of Functional Headache

**Published:** 1875-12

**Authors:** 


Treatment of Functional Headache.—For the headaches which depend on anæmia of the brain, usually resulting from nervous exhaustion, and following mental work or physical fatigue, Dr. McLane Hamilton gives tea or cocoa at the time, and recommends the following prescription:

℞. Strychniæ suph. gr. i;
Acidi phosh. dil.;
Tr. Ferri. clor. aa ʒvi.;
Aquæ camphoræ ad. ℥iv.
Sig.—A teaspoonful after eating.

Aromatic spirits of ammonia and sherry may be given several times a day with good effect. Muriate of ammonia is an invaluable remedy in these headaches, particularly in hemicrania. It should be given in very large doses—from ten grains to thirty—every hour until relief is obtained.

The *hyperæsthetic* headache demands opposite treatment, and is aggravated by stimulants. These are the cases where there are redness of the face, tense carotids, injected conjunctivæ, heat of skin, restlessness and confusion of the mental faculties. During the paroxysms the hands and feet are generally cold, and the patients suffer from sleeplessness. For these he believes bromide of sodium to be the most efficacious remedy, and next to it the bromide of calcium. He recommends:

℞. Sodii bromidi, ℥i.;
Fld. ext. ergctt, ℥i.
Aquæ camphoræ, ad. ℥iv.
Sig.—A teaspoonful every three hours, or two teaspoonfuls at night.

In these headaches, cardiac sedatives, such as aconite and veratrum viride, are of great service, and the continued use of digitalis combined with zinc is beneficial. For the headaches of inebriety he uses the monobromide of camphor, and sometimes applies leeches or cups. A common class of headaches are those depending on reflex causes, and may be called the inhibitory headaches. They are often associated with indigestion or disturbance of uterine functions, but there are some points of irritation which may readily be overlooked, such as hæmorrhoids, which often cause headache associated with great restlessness and fatigue. Its seat is usually in the frontal region, and it comes on very suddenly. The conditions occasioning these headaches are to be met by special interference and proper remedies.

In rheumatic headache the pain is superficial, and there is a diffused hyperæsthesia over the scalp which is very sensitive to touch. This is generally amenable in a few minutes to the faradaic current applied by a wire brush. In the headaches which are almost invariably associated with syphilis the inunction of mercurial ointment is often useful. The sub-occipital headache of malaria is often uncontrolled by quinine alone. The combination of arsenious acid is of great use, and the addition of a small quantity of belladonna increases its effects still more.

In neuralgia he finds the following prescription serviceable:

℞. Morph. sulph., gr. vi.;

Ext. belladonnæ;

Ext. nucis vomicæ, aa gr. xii.;

Ferri et quiniæ citrat. ʒii.

Ft. massa et divid. in pil. No. xlviii.

One three times a day.

Strychnia is of great benefit in the anæmic variety of neuralgia. Peripheral neuralgia is treated most successfully by local applications such as galvanism, chloroform, blisters, and the actual cautery. The inhalation of a few drops of nitrite of amyl often stops a severe neuralgia. Morphia injected subcutaneously is one of the best remedies; and it is perhaps even more efficacious when combined with atropine than when employed alone. Freezing the skin just in front of the ear, by ether spray, will cut short a violent attack of facial neuralgia in a few moments. In employing galvanism, the poles should be held over the nucha or lower down, and over the mastoid bone or upon both temples. In neuralgia the positive pole may be held just at the back of the ear, and the negative passed over the several branches of the fifth nerve. The faradaic current often relieves many headaches, particularly if they are diffused over the scalp, and if they are aggravated by heat to the head or pressure. Cold applications, such as bladders of ice, or the cold douche, are especially useful in headaches belonging to the hyperæsthetic variety.—*Philadelphia Medical Times.*

